# A comparative analysis of chloroplast genomes revealed the chloroplast heteroplasmy of *Artemisia annua*

**DOI:** 10.3389/fphar.2024.1466578

**Published:** 2024-08-14

**Authors:** Xiaoxia Ding, Hengyu Pan, Peiqi Shi, Siyu Zhao, Shengye Bao, Shan Zhong, Chunyan Dai, Jieting Chen, Lu Gong, Danchun Zhang, Xiaohui Qiu, Baosheng Liao, Zhihai Huang

**Affiliations:** ^1^ The Second Clinical College, Guangzhou University of Chinese Medicine, Guangzhou, China; ^2^ College of Life Science and Technology, Mudanjiang Normal University, Mudanjiang, China

**Keywords:** *Artemisia annua*, chloroplast genome, genetic diversity, strains identification, comparative analysis

## Abstract

*Artemisia annua* L. is the main source of artemisinin, an antimalarial drug. High diversity of morphological characteristics and artemisinin contents of *A. annua* has affected the stable production of artemisinin while efficient discrimination method of *A. annua* strains is not available. The complete chloroplast (cp) genomes of 38 *A. annua* strains were assembled and analyzed in this study. Phylogenetic analysis of *Artemisia* species showed that distinct intraspecific divergence occurred in *A. annua* strains. A total of 38 *A. annua* strains were divided into two distinct lineages, one lineage containing widely-distributed strains and the other lineage only containing strains from northern China. The *A. annua* cp genomes ranged from 150, 953 to 150, 974 bp and contained 131 genes, and no presence or absence variation of genes was observed. The IRs and SC junctions were located in *rps*19 and *ycf*1, respectively, without IR contraction observed. Rich sequence polymorphisms were observed among *A. annua* strains, and a total of 60 polymorphic sites representing 14 haplotypes were identified which unfolding the cpDNA heteroplasmy of *A. annua*. In conclusion, this study provided valuable resource for *A. annua* strains identification and provided new insights into the evolutionary characteristics of *A. annua*.

## 1 Introduction

*Artemisia annua* L. is the only natural source for artemisinin which is used for the treatment of malaria (Klayman, 1985; Cheong et al., 2020; Septembre-Malaterre et al., 2020; Al-Khayri et al., 2022). The selection and identification of elite germplasm of *A. annua* are critical for the high-quality, stable and low-cost production of artemisinin (Ma et al., 2014; Chen et al., 2017; Li et al., 2017; Shen et al., 2017; Ding et al., 2023). The wide range of natural distribution and high genetic heterozygosity due to self-incompatibility of pollination brought great challenges to the breeding of *A. annua* (Ma et al., 2014; Li et al., 2017; Amiryousefi et al., 2018; Ma et al., 2018). Although the genetic background of *A. annua* is relatively complex, the traits, especially artemisinin content, is correlated with their geographical distribution (Li et al., 2017). Moreover, strains of *A. annua* from southern China still had higher artemisinin content than those of northern strains under the same cultivation environment (He et al., 2022), which indicated a relative stable correlation between genetic background and geographical distribution.

Previously, a number of identification and taxonomy studies were conducted at species level of *A. annua* and its closely related species (Li et al., 2017; Shi et al., 2018; Jiao et al., 2023). *A. annua* can be efficiently identified from other *Artemisia* L. species by ITS regions (Li et al., 2017). The nuclear single nucleotide polymorphisms of 205 *Artemisia* species were used to reconstruct the phylogenetic relationships of *Artemisia* (Jiao et al., 2023). Besides, different types of molecular markers were also applied to identify different *A. annua* germplasms. He et al. (2022) applied SSR molecular markers to distinguish *A. annua* strains from different habitats. Ding et al. (2023) found that the ITS2 haplotype analysis is an ideal tool for *A. annua* strains identification based on the polymorphism of ribosomal DNA (rDNA). The cp genome is also an ideal tool for variants/strains identification and the intra-species genetic variations of cp genomes have been reported in many species (Sabir et al., 2014; Lei et al., 2016; Sun et al., 2019; Kim et al., 2020; Song et al., 2020; Xu et al., 2022). Cells of flowering plants possess high copies of their cp genome, and the copy numbers were estimated to range from 1, 900 to 50, 000 copies per cell (Bendich, 1987; Johnson and Palmer, 1989; Morley and Nielsen, 2016). Notably, heteroplasmy of organellar genome that more than one types among multiple copies of organellar genomes, was observed in individuals or even within single cells (Johnson and Palmer, 1989; Sabir et al., 2014; Lei et al., 2016; Ramsey and Mandel, 2019). Compared to DNA fragments, the whole cp genome, with relative long size, may contain sufficient variation and exhibit uniparental unisexual inheritance (Jansen and Ruhlman, 2012; Jensen and Leister, 2014; Ruhlman and Jansen, 2014), which makes it an ideal source for germplasm discrimination and genetic characteristic analysis.

In this study, a total of 38 complete cp genomes of *A. annua* were assembled and annotated. The structures, sequence polymorphisms and phylogenetic relationships of cp genomes were first in-depth analyzed and compared among *A. annua* strains. The cp genome heteroplasmy of *A. annua* was first reported in this study. Intra- and inter-individual genetic diversity of cp genomes of *A. annua* revealed in this study provided important information for the identification and evolution of *A. annua* strains.

## 2 Materials and methods

### 2.1 Materials collection, DNA extraction and PCR amplification

Thirty-five individuals of *A. annua* were cultivated in Huairou District, Beijing, from seeds collected from four countries to test and verify the polymorphic loci (Supplementary Table 1). Fresh leaves were snap-frozen with liquid nitrogen and stored at −80°C (Eppendorf, Hamburg, Germany). The total DNA of *A. annua* individuals were extracted by the modified cetyltrimethylammonium bromide (3 × CTAB) method (Allen et al., 2006), and the DNA quality and concentration were measured by electrophoresis in 1.0% agarose gel and the NanoDrop2000 ultra-micro ultraviolet spectrophotometer (Thermo Scientific, MIT, United States). Primers were designed by Primer Premier 5 (Supplementary Table 2). PCR amplification was performed on 25 μL reaction mixtures containing 2X Pro Taq Master Mix (dye plus) 12.5 μL, nuclease-free water 8.5 μL, 1.0 μL each of 10 μM forward and reverse primers, and genomic DNA 1.0 μL. The PCR reaction conditions were listed in Supplementary Table 1. The amplified products were detected by electrophoresis in 1.0% agarose gel. The synthesis of primers, and sequencing of amplification products were conducted by Sangon Biotech Guangzhou branch office.

### 2.2 WGS data collection, cp genome assembly, and annotation

WGS (whole genome sequencing) datasets of LQ-9 and HAN1 strains, and whole genome resequencing datasets of 36 individuals were obtained from previous study (Liao et al., 2022a) (Supplementary Table 3). The quality of raw sequencing data was evaluated by FastQC 0.11.5 (https://www.bioinformatics.babraham.ac.uk/projects/fastqc) and low quality bases and reads were trimmed and removed by Skewer (Jiang et al., 2014). The LQ-9 cp genome was assembled and as the reference genome for later analysis. WGS reads of LQ-9 were mapped to *A. annua* cp genome (GenBank Accession Number: MF623173) and mapped reads were extracted as cp-like reads. The extracted reads were then assembled into contigs by ABySS 2.0.0 (https://github.com/bcgsc/abyss) (Jackman et al., 2017). Finally, the cp sequence contigs were ordered and concatenated based on the collinearity with reference cp genome sequences. The initial gene annotation was conducted with plann 1.1.2 (Huang and Cronk, 2015) and then validated by BLAST and manually correction. The transport RNA (tRNA) genes were identified with tRNAscan-SE software (Lowe and Chan, 2016). Circular gene maps of the *A. annua* cp genomes were generated using Chloroplot software (Zheng et al., 2020). The cp genomes assembled in this study have been deposited in the Global Pharmacopoeia Genome Database (Liao et al., 2022b) at http://www.gpgenome.com/species/92 under the “SuperBarcodes” section.

### 2.3 Phylogenetic analysis

A total of 117 complete cp genome sequences from 38 *Artemisia* species, and one complete cp genome from *Chrysanthemum* were downloaded from the NCBI GenBank (Supplementary Table 4) for phylogenetic analysis. Among which, 115 cp genomes were reassembled since the direction of SSC regions were opposite to *A. annua* cp genome in this study. Multiple sequence alignments were performed with 156 cp genomes (Supplementary Tables 3, 4) using MAFFT 7.313 (Katoh and Standley, 2013). Maximum likelihood (ML) phylogenetic tree was performed by RAxML 8.2.11 (Stamatakis, 2014) with 1,000 bootstrap replicates under the GTR + G model. Neighbor-joining (NJ) tree was constructed using MEGA 7 (Kumar et al., 2016) under the Kimura two-parameter model with 1,000 bootstrap replicates.

### 2.4 Sequence comparison and nucleotide variation analyses

Whole cp genomes of 38 *A. annua* individuals were aligned using MAFFT 7.313 (Katoh and Standley, 2013). For rearrangement analysis, all aligned sequences were constructed with Geneious Prime program (Kearse et al., 2012). The IRscope program was used to evaluate the expansion and contraction of inverted repeat region (IR) (Amiryousefi et al., 2018). Comparative analysis of cp genomes was performed among *Artemisia* species and visualized by R. Furthermore, nucleotide diversity (Pi) was estimated by sliding window analysis using DnaSP 6.0 with a 600 bp window length and 200 bp step size (Rozas et al., 2017). High throughput sequencing (HTS) data of 38 *A. annua* individuals were aligned to the LQ-9 reference cp genome with Bowtie2 2.4.4 (Langmead and Salzberg, 2012). The variants were called and filtered using BCFtools (http://samtools.github.io/bcftools/) with a minor allele count higher than 1. False positives caused by sequencing errors were excluded by visualizing the sequencing data of mutation sites by Integrative Genomics Viewer (IGV) (Robinson et al., 2011).

## 3 Results

### 3.1 Interspecific divergence in *Artemisia* species based on cp genomes

**FIGURE 1:**
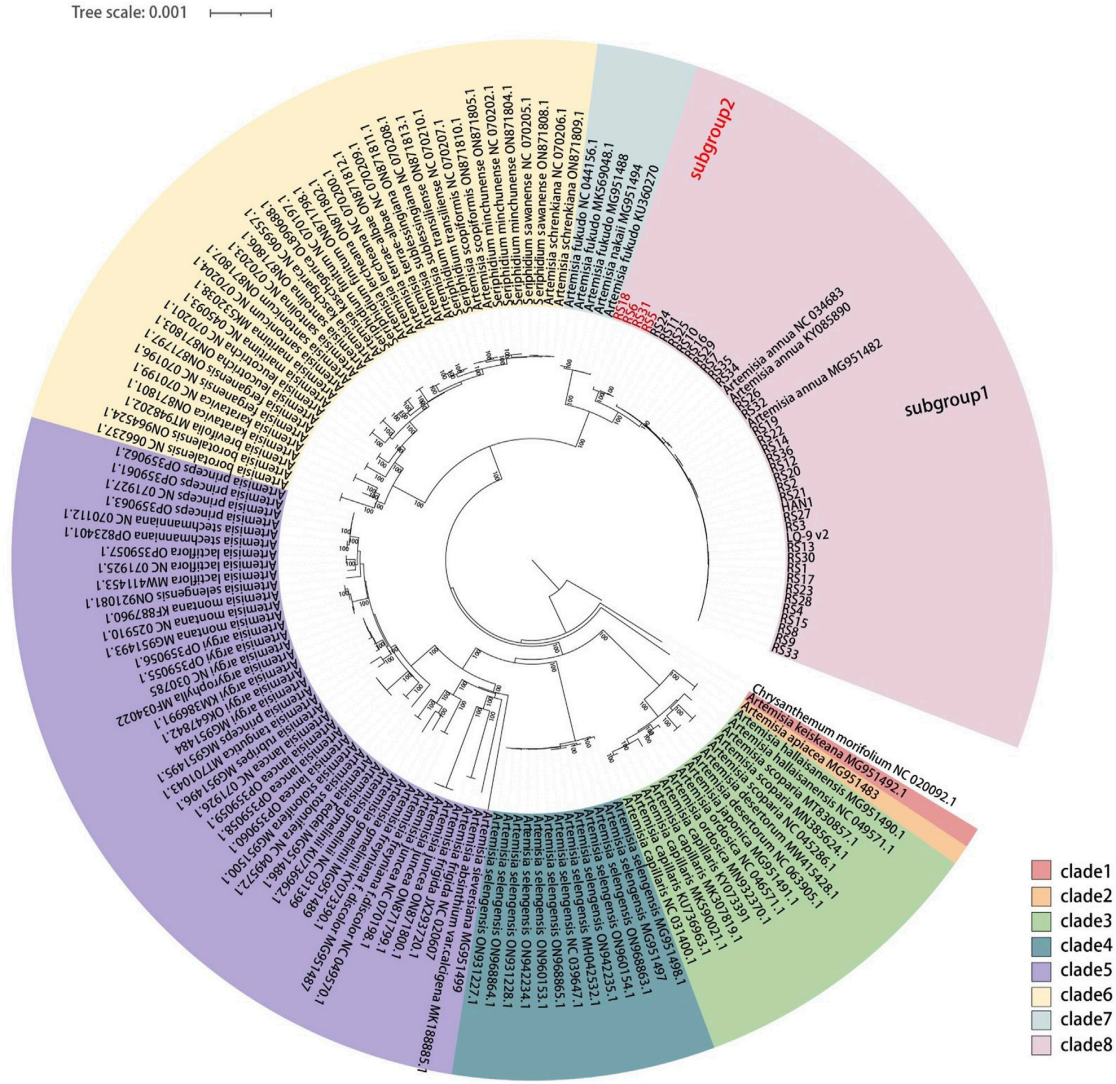
Phylogenetic tree obtained using the ML method for *Artemisia* species based on 156 complete cp genomes. Numbers above the branches indicate the ML bootstrap values.

A total of 38 complete *A. annua* cp genomes were assembled with WGS data of 38 distinct individuals released by previous study (Supplementary Table 3) (Liao et al., 2022a). To explore the phylogenetic and evolutionary relationships of *A. annua* populations, a phylogenetic tree was constructed using ML and NJ methods based on 156 complete cp genomes of 38 *Artemisia* species, and *C*. *morifolium* was included as the outgroup (Supplementary Table 4). The topological congruence between the ML and NJ phylogenetic trees was observed, with the majority of nodes exhibiting robust support values (>99%) (Figure 1; Supplementary Figure 1). Thirty-eight species were divided into two main branches, the first branch only comprising *A. keiskeana* (Figure 1, clade 1), while the second branch was further divided into seven well-supported clades (Figure 1, clades 2–8). *Seriphidium* was considered to be an independent genus (Lin et al., 2011; Haghighi et al., 2014), while this was not supported by the phylogenetic relationship of cp genomes as *Seriphidium* species were close related with *A. schrenkiana* and *A. scopiformis*. The phylogenetic tree also showed that all cp genomes from the same species clustered together, except *A. argyi* and *A. selengensis*. All *A. annua* cp genomes clustered into a same clade and shared the most recent common ancestor with *A. fukudo* and *A. nakaii*, which was consistent with the previous results of Shen et al. (2017) and Jin et al. (2023). Intriguingly, all *A. annua* strains were divided into two subgroups, forming two distinct lineages. Geographically, the first lineage (subgroup 1) containing a mixture of southern and northern strains was defined as a broad lineage, and the second (subgroup 2) was defined as a northern lineage containing only northern strains.

### 3.2 Conserved cp genome structure in *A. annua* strains

**FIGURE 2:**
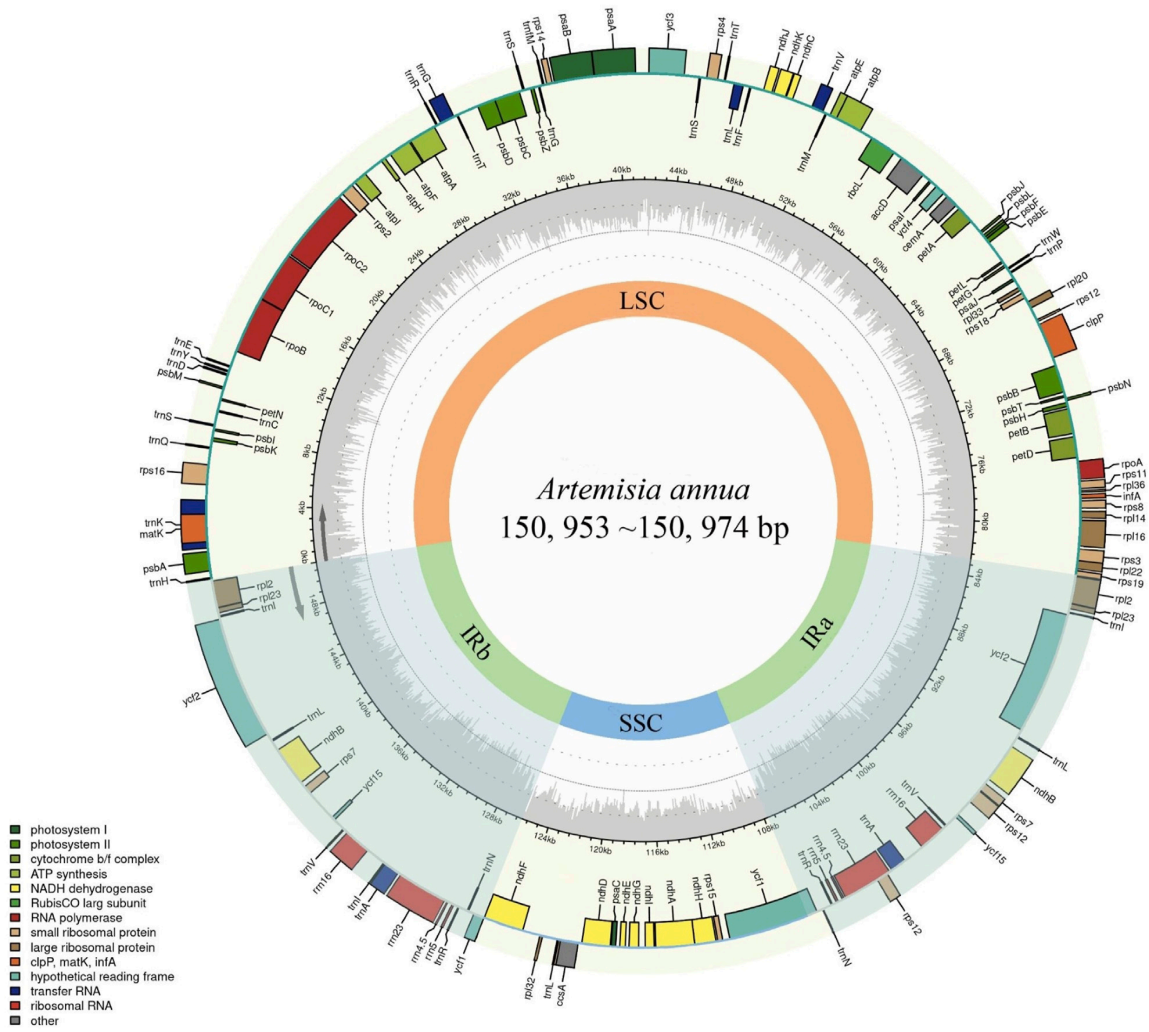
Circos plot of the *A. annua* cp genomes. Genes drawn inside the circle are transcribed in the clockwise direction, and those on the outside are transcribed in the counterclockwise direction. The genes are color-coded based on their function. The dark gray plot in the inner circle corresponds to GC content.

Thirty-eight assembled *A. annua* cp genomes were further annotated and analyzed. All cp genomes possessed the typical quadripartite structure which consisted of a large single-copy (LSC) region, a small single-copy (SSC) region, and two copies of IR regions (Figure 2). All cp genomes had lengths ranging from 150, 953 bp (LQ-9) to 150, 974 bp (RS16) with GC contents ranging from 37.47% to 37.48%. The LSC regions had lengths ranging from 82, 701 bp (RS18) to 82, 785 bp (RS24), and the SSC regions ranged from 18, 267 bp (RS24 *et al.*) to 18, 349 bp (RS18), while the lengths of IR regions were 24, 956 bp in all *A. annua* strains (Table 1), similar to the previously reported *Artemisia* cp genomes (Shen et al., 2017; Kim et al., 2020; Jin et al., 2023). Gene annotation showed that all cp genomes contained 131 genes, including 87 protein-coding genes, 36 tRNA genes and eight ribosomal RNA (rRNA) genes (Table 1). Of these, 17 genes (seven protein-coding genes, six tRNA genes and four rRNA genes) were duplicated in the IR regions in all *A. annua* strains (Supplementary Table 5). In addition, 17 intron-containing genes were identified, of which 15 contained one intron and two contained two introns (Supplementary Table 5).

**TABLE 1:** Features of 38 *A. annua* cp genomes.

Sample	Genome size (bp)	LSC length (bp)	SSC length (bp)	IR length (bp)	CDS length (bp)	Number of genes	Number of protein-coding genes	Number of tRNA genes	Number of rRNA genes	Overall GC (%)
LQ-9	150, 953	82, 774	18, 267	24, 956	79, 236	131	87	36	8	37.48
HAN1	150, 956	82, 777	18, 267	24, 956	79, 236	131	87	36	8	37.48
RS1	150, 955	82, 776	18, 267	24, 956	79, 236	131	87	36	8	37.48
RS2	150, 956	82, 777	18, 267	24, 956	79, 236	131	87	36	8	37.48
RS3	150, 957	82, 778	18, 267	24, 956	79, 236	131	87	36	8	37.48
RS4	150, 955	82, 776	18, 267	24, 956	79, 236	131	87	36	8	37.48
RS5	150, 959	82, 706	18, 341	24, 956	79, 236	131	87	36	8	37.47
RS6	150, 957	82, 704	18, 341	24, 956	79, 237	131	87	36	8	37.48
RS7	150, 963	82, 766	18, 285	24, 956	79, 236	131	87	36	8	37.48
RS8	150, 955	82, 776	18, 267	24, 956	79, 236	131	87	36	8	37.48
RS9	150, 955	82, 776	18, 267	24, 956	79, 236	131	87	36	8	37.48
RS10	150, 972	82, 776	18, 284	24, 956	79, 236	131	87	36	8	37.48
RS11	150, 960	82, 763	18, 285	24, 956	79, 236	131	87	36	8	37.48
RS12	150, 963	82, 784	18, 267	24, 956	79, 236	131	87	36	8	37.48
RS13	150, 955	82, 776	18, 267	24, 956	79, 236	131	87	36	8	37.48
RS14	150, 958	82, 779	18, 267	24, 956	79, 236	131	87	36	8	37.48
RS15	150, 955	82, 776	18, 267	24, 956	79, 236	131	87	36	8	37.48
RS16	150, 974	82, 778	18, 284	24, 956	79, 236	131	87	36	8	37.48
RS17	150, 955	82, 776	18, 267	24, 956	79, 236	131	87	36	8	37.48
RS18	150, 962	82, 701	18, 349	24, 956	79, 236	131	87	36	8	37.48
RS19	150, 957	82, 778	18, 267	24, 956	79, 236	131	87	36	8	37.48
RS20	150, 959	82, 780	18, 267	24, 956	79, 236	131	87	36	8	37.48
RS21	150, 957	82, 778	18, 267	24, 956	79, 236	131	87	36	8	37.48
RS22	150, 958	82, 779	18, 267	24, 956	79, 236	131	87	36	8	37.48
RS23	150, 955	82, 776	18, 267	24, 956	79, 236	131	87	36	8	37.48
RS24	150, 964	82, 785	18, 267	24, 956	79, 248	131	87	36	8	37.48
RS25	150, 972	82, 776	18, 284	24, 956	79, 236	131	87	36	8	37.48
RS26	150, 955	82, 776	18, 267	24, 956	79, 236	131	87	36	8	37.48
RS27	150, 956	82, 777	18, 267	24, 956	79, 236	131	87	36	8	37.48
RS28	150, 955	82, 776	18, 267	24, 956	79, 236	131	87	36	8	37.48
RS29	150, 963	82, 766	18, 285	24, 956	79, 236	131	87	36	8	37.48
RS30	150, 955	82, 776	18, 267	24, 956	79, 236	131	87	36	8	37.48
RS31	150, 959	82, 706	18, 341	24, 956	79, 236	131	87	36	8	37.47
RS32	150, 955	82, 776	18, 267	24, 956	79, 236	131	87	36	8	37.48
RS33	150, 955	82, 776	18, 267	24, 956	79, 236	131	87	36	8	37.48
RS34	150, 969	82, 772	18, 285	24, 956	79, 236	131	87	36	8	37.48
RS35	150, 968	82, 771	18, 285	24, 956	79, 236	131	87	36	8	37.48
RS36	150, 958	82, 779	18, 267	24, 956	79, 236	131	87	36	8	37.48

The IR border structure was conserved in *A. annua* that no IR contraction was observed. In each *A. annua* individual, the junctions between IRs and LSC and SSC were flanked by *rps*19 and *ycf*1, respectively (Supplementary Figure 2), in concordance with other *Artemisia* species (Kim et al., 2020; Jin et al., 2023). Comparison of genome structure showed that *A. annua* cp genomes were highly conserved, and no significant gene rearrangements were observed (Supplementary Figure 3). On the whole, the *A. annua* cp genomes were structural conserved.

### 3.3 Sequence polymorphisms in *A. annua* cp genomes

**FIGURE 3:**
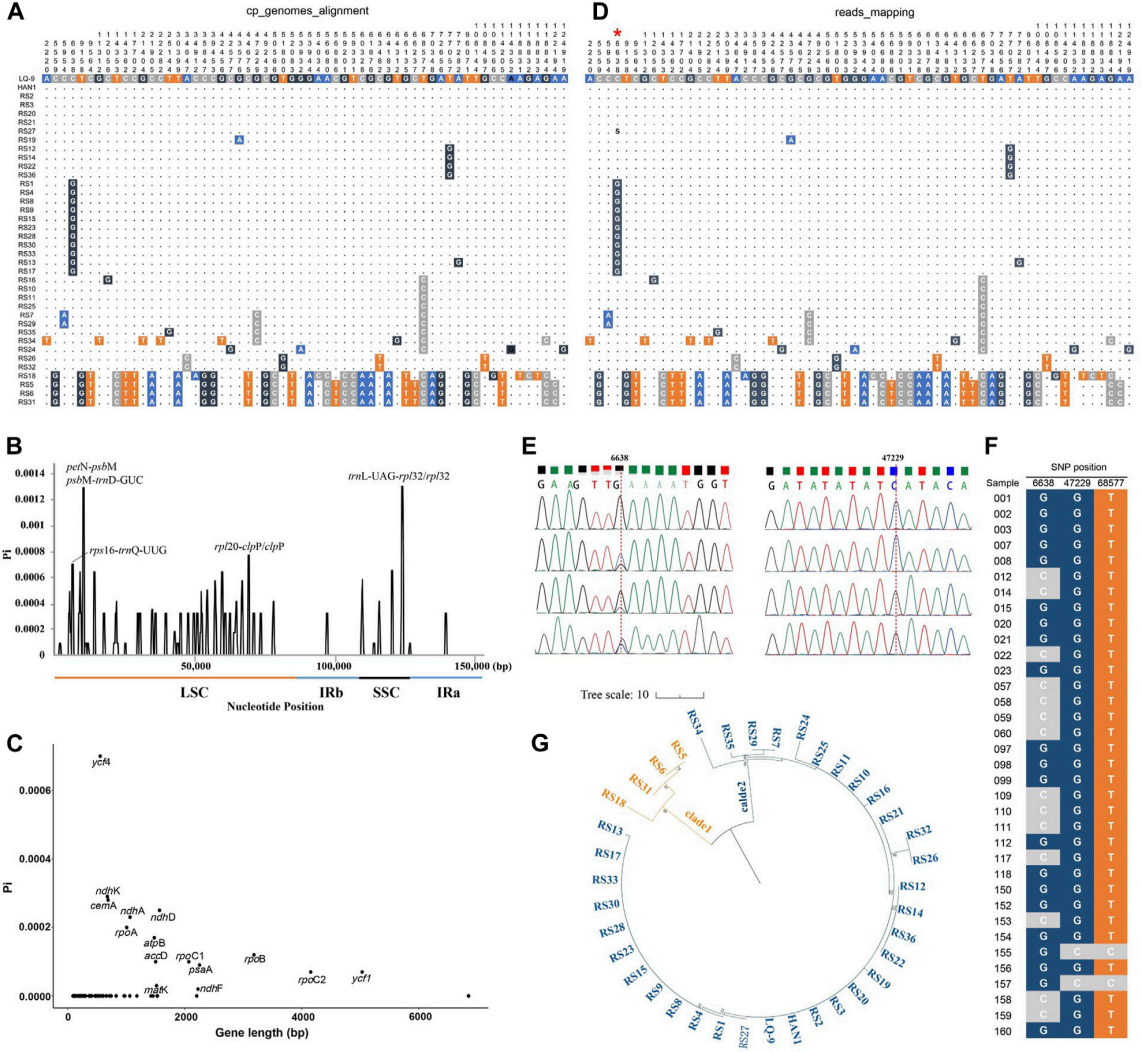
Variation of *A. annua* cp genomes. **(A)** Polymorphism analysis based on 38 *A. annua* global sequence alignment; **(B)** Nucleotide diversity of 38 *A. annua* cp genomes; **(C)** Nucleotide diversity of protein-coding genes; **(D)** Polymorphism analysis based on WGS reads mapping, red asterisk represents the pSNP site; **(E)** Partial peak diagram of Sanger sequencing of polymorphic sites 6,638 and 47,229, respectively; **(F)** Polymorphic sites confirmed by Sanger sequencing; **(G)** NJ tree constructed based on 60 polymorphic sites.

**FIGURE 4:**
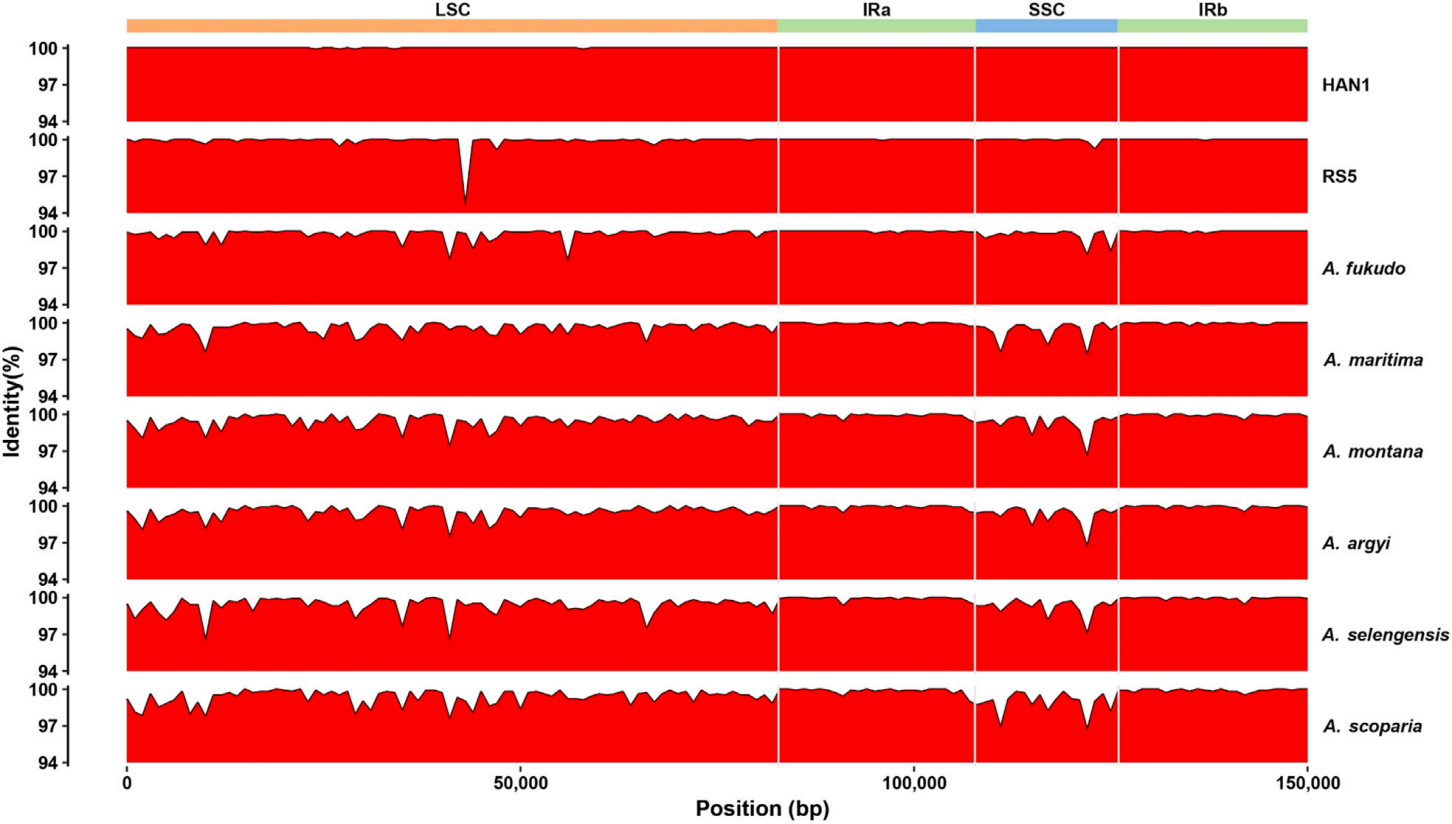
Identity plot based on the whole cp genome comparison of *Artemisia* species.

In this study, the intraspecific polymorphisms of *A. annua* cp genomes were assessed in inter-individual levels. Complete cp genomes of 38 *A. annua* individuals were aligned using the LQ-9 as the reference genome. In total, 60 polymorphic sites were identified after excluding false positives caused by sequencing errors, including 19 singleton variable sites and 41 parsimony informative sites, among which, 48 SNPs were identified in LSC, 11 in SSC and one in IR regions, respectively (Figure 3A; Table 2). The SSC regions were found to have the highest polymorphisms with a SNP site density of 8.8/10 kb, followed by LSC regions (5.3/10 kb), and the SNP density of IRs regions was only 0.4/10 kb (Table 2), revealing that the IR regions were more conserved than the single-copy regions, which is consistent with the comparative analysis of the whole cp genome of *Artemisia* species (Figure 4). Furthermore, the Pi values of 38 *A. annua* cp genomes were calculated using DnaSP v6.0, and the Pi values ranged from 0 to 0.0013 with an average of 0.00007. Based on DNA polymorphisms, seven highly diverged regions (Pi > 0.0007) were identified, including *rps*16-*trn*Q-UUG, *pet*N-*psb*M, *psb*M*-trn*D*-*GUC, *rpl*20*-clp*P, *clp*P, *trn*L*-*UAG*-rpl*32 and *rpl*32 (Figure 3B), similar results have been observed in other *Artemisia* species (Shen et al., 2017; Kim et al., 2020; Jin et al., 2023). Fifteen protein-coding genes were found to containing variations, of which the *ycf*4 gene had the highest Pi value (0.0007) and the *ndh*F gene had the lowest Pi value (0.000002) (Figure 3C; Supplementary Table 6).

**TABLE 2:** Number of SNPs in each region of cp genomes combing 38 *A. annua* individuals.

Region	Length	SNP count	Transversion	Transition	SNP density/10 kb
LSC	82, 774	48	31	17	5.3
SSC	18, 267	11	7	4	8.8
IR	49, 912	1	1	0	0.4

In addition, the WGS data of 38 individuals were aligned to LQ-9 reference genome with Bowtie2 for polymorphic sites validation. All 60 polymorphic sites were supported by mapped reads (Figures 3A, D). Notably, most of the polymorphic sites were single nucleotide polymorphisms (SNPs) between *A. annua* individuals, but partial single nucleotide polymorphism (pSNP) which contained more than one alternative bases in one site defined by James et al. (2009) were also found within individuals (Figure 3D). The polymorphic loci were confirmed by Sanger sequencing using the total DNA of 35 *A. annua* individuals (Figure 3F; Supplementary Table 1). In the Sanger sequencing results, the pSNP site generally contained multiple nested-peak which were identified as degenerate bases, and the SNP site showed clean single-peak representing complete base mutations (Figure 3E).

A total of 14 haplotypes were found in 38 *A. annua* individuals, which can be further divided into two distinct lineages as shown above (Figure 1; Figure 3G). Germplasm resources collected from the same location clustered together excluding Gansu, Hubei, and Xizang populations, but strains from different populations were also observed to have the same haplotype. Besides, polymorphism analysis showed that the subgroup 2 (the northern lineage) exhibited more unique polymorphic sites than the subgroup 1 (the broad lineage) and they shared only two polymorphic sites (Table 3). Other *Artemisia* species shared fifteen and four polymorphic sites with the northern lineage and the broad lineage, respectively (Table 3; Supplementary Figure 4). We proposed that the broad lineage may be the stable and specific evolutionary lineage during the speciation of *A. annua*, and the northern lineage may be the post-forming lineage. The above results showed that *A. annua* cp genomes were highly polymorphic, and the cp variants dataset can be used as the reference dataset for the identification and evolutionary analysis of *A. annua* strains.

**TABLE 3:** Comparison of variations between 38 *A. annua* strains and other *Artemisia* species.

	Variation types	Number of polymorphic sites
Subgroup 1 vs. Subgroup 2	Unique (Subgroup 1)	22
	Unique (Subgroup 2)	36
	Common	2
Subgroup 1 vs. Other *Artemisia*	Unique (Subgroup 1)	20
	Common	4
Subgroup 2 vs. Other *Artemisia*	Unique (Subgroup 2)	23
	Common	15

## 4 Discussion

In this study, the phylogenetic relationships, structures, sequence polymorphisms of *A. annua* cp genomes were analyzed. All *A. annua* cp genomes showed highly conserved in structures, gene numbers and gene order. Minor differences were observed in genome sizes, but the lengths of IR regions were identical in all strains without contraction, similar to the observation in other *Artemisia* species (Shen et al., 2017; Kim et al., 2020; Jin et al., 2023). CpDNA is multicopy and abundant in plant (Bendich, 1987; McPherson et al., 2013; Malé et al., 2014; Morley and Nielsen, 2016), and were thought to be very conserved, as each species is typically characterized by a single cpDNA type. Recently, some genome-based studies have focused on population-level resolution and a single individual per species, and intraspecific genetic variations were detected in many species (Cui et al., 2020; Chen et al., 2022; Huang et al., 2022). Similarly, high sequence polymorphisms were found among *A. annua* populations. Distinct intraspecific divergence was observed, in which a more complex population structure was assumed in broad lineage due to their multiple maternal lineages and relatively higher number of haplotypes.

Although multiple copies and heteroplasmy of organelle genomes had been discovered over a century, the phenomenon of heteroplasmy was often overlooked in previous studies (Bendich, 1987; Ramsey and Mandel, 2019). The mitochondrial heteroplasmy in humans has been studied extensively (Li et al., 2010; Jiang et al., 2023) as its occurrence is strongly related with mitochondrial diseases (Wallace, 1992; Wallace, 1994; Chinnery and Turnbull, 1999; Chinnery et al., 2002). Besides, the heteroplasmy characteristic of mitochondria have also been used as genetic markers in molecular identification (Salas et al., 2001; Salas et al., 2005; Jiang et al., 2023). Plants possess high copy numbers of cp genomes, and polymorphisms of cp genomes are common at the level of population or species (Johnson and Palmer, 1989; Kim et al., 2020; Song et al., 2020; Xu et al., 2022). However, intra and inter-individual heteroplasmy of cp genomes are rarely reported (Fitter et al., 1996; García et al., 2004; Frey et al., 2005). In this study, the heteroplasmy of *A. annua* cp genomes were first discovered and verified, which may provide an important genetic basis for the accurate identification and evolutionary analysis of *A. annua* strains in the future.

This study represents the first in-depth understanding of *A. annua* cp genomes, and the rich maternal haplotypes composition can be used as the reference dataset for strains identification and breeding of *A. annua*. As cp genome is inherited matrilineally and relatively conserved, the combination of cp genome haplotypes and trait-related loci from nuclear genome should be further applied to *A. annua* strains screening in the future.

## 5 Conclusion

In the present study, the phylogenetic relationships, structures, sequence polymorphisms of *A. annua* cp genomes were characterized. Phylogenetic analysis of *Artemisia* species showed that intraspecific divergence occurred during the speciation of *A. annua*, forming two distinct lineages, the broad lineage and the northern lineage. The structures of *A. annua* cp genomes were extremely conserved without IR contraction and gene rearrangements observed. Rich sequence polymorphisms were observed among *A. annua* strains, and cpDNA heteroplasmy has been first reported and verified in *A. annua*. A total of 60 polymorphic sites were identified by global sequence alignment and WGS reads mapping, which can be further divided into 14 haplotypes, representing the cp variants dataset of *A. annua*. Our work provides important information for the identification and evolution of *A. annua* strains.

## Data Availability

The WGS data and assembled cp genome data were deposited in the Global Pharmacopoeia Genome Database at http://www.gpgenome.com/species/92 under the “SuperBarcodes” section. Data supporting the findings of this work are available within the paper and its supplemental information files. The datasets generated and analyzed during the study are available from the corresponding author upon reasonable request.

## Abbreviations

cp, chloroplast; rDNA, ribosomal DNA; WGS, whole genome sequencing; tRNA, transport RNA; ML, maximum likelihood; NJ, neighbor-joining; IR, inverted repeat region; Pi, nucleotide diversity; HTS, high-throughput sequencing; IGV, Integrative Genomics Viewer; LSC, large single-copy region; SSC, small single-copy region; rRNA, ribosomal RNA; CDS, coding sequence; SNPs, single nucleotide polymorphisms; pSNP, partial single nucleotide polymorphism.
