# Supplementary material for: A comparative analysis of chloroplast genomes revealed the chloroplast heteroplasmy of Artemisia annua
Source: Front Pharmacol. 2024 Aug 14;15:1466578. doi: 10.3389/fphar.2024.1466578 (PMC11349571; doi:10.3389/fphar.2024.1466578)
Supplement: Supplementary file 1 [file Table1.pdf]

Table S1 Sanger sequencing samples of *A. annua*

Sample ID	Strain	Sample	Location
H001	JL1	D-JL1-4	Yongji County, Jilin Province
H002	HAN1	D-HAN1 1-3	Qiongzhong, Hainan Province
H003	LN2	D-LN2-1	Huludao, Liaoning Province
H007	GX3	D-GX3 1-1	Guilin, Guangxi Province
H008	GX1	D-GX1-2	Hezhou Geological Museum, Guangxi Province
H012	HU1	D-HU1 1-3	Harbin, Heilongjiang Province
H014	JL1	D-JL1-1	Yongji County, Jilin Province
H015	JL1	D-JL1-2	Yongji County, Jilin Province
H020	HLJ1	D-HLJ1 1-1	Yichun, Heilongjiang Province
H021	HLJ1	D-HLJ1 1-2	Yichun, Heilongjiang Province
H022	HLJ1	D-HLJ1 1-3	Yichun, Heilongjiang Province
H023	HLJ1	D-HLJ1 1-4	Yichun, Heilongjiang Province
H057	HEN1	D-HEN1-1	Tongbo County, Henan Province
H058	HEN1	D-HEN1-2	Tongbo County, Henan Province
H059	HEN1	D-HEN1-3	Tongbo County, Henan Province
H060	HEN1	D-HEN1-4	Tongbo County, Henan Province
H097	CQ1	D-CQ1 1-1	Chongqing
H098	CQ1	D-CQ1 1-2	Chongqing
H099	CQ1	D-CQ1 1-3	Chongqing
H109	HUB1	D-HUB1-1	Enshi, Hubei Province
H110	HUB1	D-HUB1-2	Enshi, Hubei Province
H111	HUB1	D-HUB1-3	Enshi, Hubei Province
H112	HUB1	D-HUB1-4	Enshi, Hubei Province
H117	GX1	D-GX1-1	Hezhou Geological Museum, Guangxi Province
H118	GX1	D-GX1-3	Hezhou Geological Museum, Guangxi Province
H150	FR	D-FR1-1	France
H152	FR	D-FR1-3	France
H153	FR	D-FR1-4	France
H154	SW	D-SW-1	Switzerland
H155	FR	D-SW-2	Switzerland
H156	SW	D-SW-3	Switzerland
H157	SW	D-SW-4	Switzerland
H158	TH	D-TH-1	Thailand
H159	TH	D-TH-3	Thailand
H160	TH	D-TH-4	Thailand

Table S2 Primers used for variable regions amplification

**Primer name**	**Sequence (5'-3')**	**PCR amplification conditions**	**Amplification length (bp)**
6638-F	TTGCTTTCTACCACATCGTTTT	Initial denaturing at 98 ℃ for 30 sec; follow by 15 cycles of denaturation at 98 ℃ for 10 sec, annealing at 57 ℃ for 5 ses, and elongation at 72 ℃ for 4 ses, with a final extension at 72 ℃ for 1 min.	543
6638-R	TTATTTGCTTTGCATCATTGAC		
47229-F	TCAAGTCCCTCTATCCCCAAA	Initial denaturing at 98 ℃ for 30 sec; follow by 15 cycles of denaturation at 98 ℃ for 10 sec, annealing at 58 ℃ for 5 ses, and elongation at 72 ℃ for 4 ses, with a final extension at 72 ℃ for 1 min.	308
47229-R	CAAAGCCTCATCCAGGTCCTA		
68577-F	AACGAGTCGCACATACACCC	Initial denaturing at 98 ℃ for 30 sec; follow by 15 cycles of denaturation at 98 ℃ for 10 sec, annealing at 58 ℃ for 5 ses, and elongation at 72 ℃ for 4 ses, with a final extension at 72 ℃ for 1 min.	335
68577-R	GTCAGCAACAGAAGCCCAAG		

Table S3 HTS data of 38 *A. annua* samples

Sample ID	Strain	Location	Longitude	Latitude	Artemisinin content
LQ-9	LQ-9	Qiongzhong, Hainan province	E159°50′47.14″	N36°43′36.30″	0.11±0.01
HAN1	HAN1	Qiongzhong, Hainan province	E109°46′23.16″	N19°02′29.00″	1.05±0.09
RS5	BJ1	Beijing Badaling National Forest Park	E116°0′33.40″	N40°21′19.10″	0.14±0.02
RS31	BJ1	Beijing Badaling National Forest Park	E116°0′33.40″	N40°21′19.10″	0.14±0.02
RS6	BJ1	Beijing Badaling National Forest Park	E116°0′33.40″	N40°21′19.10″	0.14±0.02
RS11	GS1	Longnan, Gansu Province	E104°35'23.76″	N33°37'39.85″	0.03±0.06
RS18	GS1	Longnan, Gansu Province	E104°35'23.76″	N33°37'39.85″	0.03±0.06
RS19	GS1	Longnan, Gansu Province	E104°35'23.76″	N33°37'39.85″	0.03±0.06
RS34	GS1	Longnan, Gansu Province	E104°35'23.76″	N33°37'39.85″	0.03±0.06
RS35	GS1	Longnan, Gansu Province	E104°35'23.76″	N33°37'39.85″	0.03±0.06
RS1	GX1	Hezhou Geological Museum, Guangxi Province	E111°30′26.40″	N24°26′39.63″	0.58±0.11
RS15	GX1	Hezhou Geological Museum, Guangxi Province	E111°30′26.40″	N24°26′39.63″	0.58±0.11
RS28	GX1	Hezhou Geological Museum, Guangxi Province	E111°30′26.40″	N24°26′39.63″	0.58±0.11
RS7	GZ1	Songtao County, Guizhou Province	E109°11′43.37″	N28°10′53.91″	0.64±0.04
RS29	GZ1	Songtao County, Guizhou Province	E109°11′43.37″	N28°10′53.91″	0.64±0.04
RS3	HAN1	Qiongzhong, Hainan province	E109°46′23.16″	N19°02′29.00″	0.82±0.09
RS27	HAN1	Qiongzhong, Hainan province	E109°46′23.16″	N19°02′29.00″	0.82±0.09
RS14	HEN1	Tongbo County, Henan Province	E116°0′33.40″	N40°21′19.10″	0.34±0.10
RS22	HEN1	Tongbo County, Henan Province	E116°0′33.40″	N40°21′19.10″	0.34±0.10
RS16	HUB1	Enshi, Hubei Province	E109°26′29.40″	N30°20′41.17″	0.53±0.18
RS20	HUB1	Enshi, Hubei Province	E109°26′29.40″	N30°20′41.17″	0.53±0.18
RS10	HUN1	Huaihua, Hunan Province	E109°28′17.02″	N27°26′37.05″	0.81±0.14
RS25	HUN1	Huaihua, Hunan Province	E109°28′17.02″	N27°26′37.05″	0.81±0.14
RS26	JL1	Yongji County, Jilin Province	E126°0′26.22″	N43°47′15.14″	0.03±0.03
RS32	JL1	Yongji County, Jilin Province	E126°0′26.22″	N43°47′15.14″	0.03±0.03
RS12	JS	Hongshan National Archaeological Site Park, Wuxi, Jiangsu Province	E120°30'36.93″	N31°29'24.05″	0.50±0.12
RS36	JS	Hongshan National Archaeological Site Park, Wuxi, Jiangsu Province	E120°30'36.93″	N31°29'24.05″	0.50±0.12
RS23	XZ1	Red Sun rural Credit Cooperative, Linzhi Mirin County, Xizang Province	E94°21'16.04″	N29°39'25.69″	0.26±0.10
RS24	XZ2	Red Sun rural Credit Cooperative, Linzhi Mirin County, Xizang Province	E94°21'16.04″	N29°39'25.69″	0.26±0.10
RS2	YN2	Xinping County, Yunnan Province	E101°59′19.36″	N24°04′22.27″	0.51±0.14
RS21	YN2	Xinping County, Yunnan Province	E101°59′19.36″	N24°04′22.27″	0.51±0.14
RS4	YQ1	Guangzhou, Guangdong Province	E116°25′18.50″	N39°56′13.38″	1.09±0.17
RS17	YQ1	Guangzhou, Guangdong Province	E116°25′18.50″	N39°56′13.38″	1.09±0.17
RS30	YQ1	Guangzhou, Guangdong Province	E116°25′18.50″	N39°56′13.38″	1.09±0.17
RS13	YQ2	Changsha, Hunan Province	E116°25′18.50″	N39°56′13.38″	1.41±0.09
RS33	YQ2	Changsha, Hunan Province	E116°25′18.50″	N39°56′13.38″	1.41±0.09
RS8	YQ7	Nanning, Guangxi Province	E116°25′18.50″	N39°56′13.38″	1.55±0.27
RS9	YQ7	Nanning, Guangxi Province	E116°25′18.50″	N39°56′13.38″	1.55±0.27

Table S4 Species used in phylogenetic analysis

Species	GenBank accession number	Note
Artemisia absinthium var. *calcigena*	MK188885.1	
Artemisia annua	NC_034683.1	
Artemisia annua	MG951482.1	
Artemisia annua	KY085890.1	
Artemisia apiacea	MG951483.1	
Artemisia argyi	OP359056.1	
Artemisia argyi	OP359055.1	
Artemisia argyi	NC_030785.1	
Artemisia argyi	KM386991.1	
Artemisia argyi	OK647842.1	
Artemisia argyi	MG951484.1	
Artemisia argyrophylla	MF034022.1	
Artemisia borotalensis	NC_066237.1	
Artemisia borotalensis	ON964524.1	
Artemisia brevifolia	MT948202.1	
Artemisia capillaris	KY073391.1	
Artemisia capillaris	NC_031400.1	
Artemisia capillaris	MK307819.1	
Artemisia capillaris	MK590021.1	
Artemisia capillaris	KU736963.1	
Artemisia desertorum	NC_063905.1	
Artemisia desertorum	MW415428.1	
Artemisia feddei	MG951486.1	
Artemisia ferganensis	NC_070196.1	
Artemisia ferganensis	ON871797.1	
Artemisia freyniana f. *discolor*	NC_049570.1	
Artemisia freyniana f. *discolor*	MG951487.1	
Artemisia frigida	NC_020607.1	
Artemisia frigida	JX293720.1	
Artemisia fukudo	KU360270.1	
Artemisia fukudo	NC_044156.1	
Artemisia fukudo	MK569048.1	
Artemisia fukudo	MG951488.1	
Artemisia gmelinii	NC_031399.1	
Artemisia gmelinii	KY073390.1	
Artemisia gmelinii	KU736962.1	
Artemisia gmelinii	MG951489.1	
Artemisia hallaisanensis	NC_049571.1	
Artemisia hallaisanensis	MG951490.1	
Artemisia japonica	MG951491.1	
Artemisia juncea	NC_070198.1	
Artemisia juncea	ON871800.1	

Artemisia juncea	ON871799.1
Artemisia karatavica	NC_070199.1
Artemisia karatavica	ON871801.1
Artemisia kaschgarica	NC_069557.1
Artemisia kaschgarica	OL890688.1
Artemisia keiskeana	MG951492.1
Artemisia lactiflora	NC_071925.1
Artemisia lactiflora	OP359057.1
Artemisia lactiflora	MW411453.1
Artemisia lancea	NC_071926.1
Artemisia lancea	OP359060.1
Artemisia lancea	OP359059.1
Artemisia lancea	OP359058.1
Artemisia lercheana	NC_070200.1
Artemisia lercheana	ON871802.1
Artemisia leucotricha	NC_070201.1
Artemisia leucotricha	ON871803.1
Artemisia maritima	NC_045093.1
Artemisia maritima	MK532038.1
Artemisia montana	NC_025910.1
Artemisia montana	KF887960.1
Artemisia montana	MG951493.1
Artemisia nakaii	MG951494.1
Artemisia ordosica	MN932370.1
Artemisia ordosica	NC_046571.1
Artemisia princeps	NC_071927.1
Artemisia princeps	OP359063.1
Artemisia princeps	OP359062.1
Artemisia princeps	OP359061.1
Artemisia princeps	MG951495.1
Artemisia rubripes	MG951496.1
Artemisia santolina	NC_070203.1
Artemisia santolina	ON871806.1
Artemisia santonicum	NC_070204.1
Artemisia santonicum	ON871807.1
Artemisia schrenkiana	NC_070206.1
Artemisia schrenkiana	ON871809.1
Artemisia scoparia	NC_045286.1
Artemisia scoparia	MN385624.1
Artemisia scoparia	MT830857.1
Artemisia scopiformis	NC_070207.1
Artemisia scopiformis	ON871810.1
Artemisia selengensis	ON968865.1
Artemisia selengensis	ON968864.1

Artemisia selengensis	ON968863.1	
Artemisia selengensis	ON960154.1	
Artemisia selengensis	ON960153.1	
Artemisia selengensis	ON942235.1	
Artemisia selengensis	ON942234.1	
Artemisia selengensis	ON931228.1	
Artemisia selengensis	ON931227.1	
Artemisia selengensis	ON921081.1	
Artemisia selengensis	NC_039647.1	
Artemisia selengensis	MH042532.1	
Artemisia selengensis	MG951498.1	
Artemisia selengensis	MG951497.1	
Artemisia sieversiana	MG951499.1	
Artemisia stechmanniana	NC_070112.1	
Artemisia stechmanniana	OP823401.1	
Artemisia stolonifera	NC_049572.1	
Artemisia stolonifera	MG951500.1	
Artemisia sublessingiana	NC_070208.1	
Artemisia sublessingiana	ON871811.1	
Artemisia tangutica	MT701043.1	
Artemisia terrae-albae	NC_070209.1	
Artemisia terrae-albae	ON871812.1	
Seriphidium finitum	NC_070197.1	
Seriphidium finitum	ON871798.1	
Seriphidium minchunense	NC_070202.1	
Seriphidium minchunense	ON871805.1	
Seriphidium minchunense	ON871804.1	
Seriphidium sawanense	NC_070205.1	
Seriphidium sawanense	ON871808.1	
Seriphidium transiliense	NC_070210.1	
Seriphidium transiliense	ON871813.1	
Chrysanthemum morifolium	NC_020092.1	Outgroup

Table S5 Gene contents in *A. annua* cp genomes

**Category for genes**	**Group of genes**	**Name of genes**	**Gene number**
Photosynthesis	Subunits of photosystem I	*psa*A, *psa*B, *psa*C, *psa*I, *psa*J, *ycf*3[**], *ycf*4	7
	Subunits of photosystem II	*psb*A, *psb*B, *psb*C, *psb*D, *psb*E, *psb*F, *psb*H, *psb*I, *psb*J, *psb*K, *psb*L, *psb*M, *psb*N, *psb*T, *psb*Z	15
	Subunits of ATP synthase	*atp*A, *atp*B, *atp*E, *atp*F[*], *atp*H, *atp*I	6
	Subunits of cytochrome b/f complex	*pet*N, *pet*A, *pet*L, *pet*G, *pet*B[*], *pet*D[*]	6
	Subunits of NADH dehydrogenase	*ndh*A[*], *ndh*B[a*], *ndh*C, *ndh*D, *ndh*E, *ndh*F, *ndh*G, *ndh*H, *ndh*I, *ndh*J, *ndh*K	12
Self-replication	Large subunit of rubisco	*rbc*L	1
	Subunits of RNA polymerase	*rpo*A, *rpo*B, *rpo*C1[*], *rpo*C2,	4
	Large ribosomal subunit	*rpl*2[a*], *rpl*14, *rpl*16[*], *rpl*20, *rpl*22, *rpl*23[a], *rpl*32, *rpl*33, *rpl*36	11
	Small ribosomal subunit	*rps*2, *rps*3, *rps*4, *rps*7[a], *rps*8, *rps*11, *rps*12[a*], *rps*14, *rps*15, *rps*16[*], *rps*18, *rps*19	14
	Ribosomal RNAs	*rrn*4.5[a], *rrn*5[a], *rrn*16[a], *rrn*23[a]	8
	Transfer RNAs	*trn*H-GUG, *trn*K-UUU[*], *trn*Q-UUG, *trn*S-GCU, *trn*C-GCA, *trn*D-GUC, *trn*Y-GUA, *trn*E-UUC, *trn*R-UCU, *trn*G-UCC[*], *trn*T-GGU, *trn*S-UGA, *trn*G-UCC, *trn*M-CAU, *trn*S-GGA, *trn*T-UGU, *trn*L-UAA[*], *trn*F-GAA, *trn*V-UAC[*], *trn*W-CCA, *trn*P-UGG, *trn*I-CAU[a], *trn*L-CAA[a], *trn*V-GAC[a], *trn*I-GAU, *trn*A-UGC[a*], *trn*R-ACG[a], *trn*N-GUU[a], *trn*L-UAG, *trnf*M-CAU	36
Biosynthesis	Translational initiation factor	*inf*A	1
	c-type cytochrome synthesis gene	*ccs*A	1
	Envelope membrane protein	*cem*A	1
	Subunit of Acetyl-CoA-carboxylase	*acc*D	1
	Protease	*clp*P[**]	1
	Maturase	*mat*K	1
Unknown function	Conserved open reading frames	*ycf*1[a], *ycf*2[a], *ycf*15	5
	Pseudo genes	*ycf*15	1

Gene with one intron, [**]Gene with two introns, [a]Gene with two copies

Table S6 Nucleotide diversity of protein-coding genes

**Gene**	**Length of alignment (bp)**	**Avg. pairwise similarity (%)**	**Identical sites (%)**	**π (10^{-3})**	**Total variable sites**	**Singleton sites**	**Parsimony informative sites**
*ycf*4	555	99.99	99.64	0.7	2	0	2
*ndh*K	678	99.99	99.85	0.29	1	0	1
*cem*A	690	99.99	99.85	0.28	1	0	1
*ndh*D	1569	99.99	99.74	0.25	4	3	1
*ndh*A	1065	99.99	99.81	0.23	2	1	1
*rpo*A	1008	99.99	99.80	0.2	2	1	1
*atp*B	1479	99.99	99.86	0.17	2	1	1
*rpo*B	3183	99.99	99.94	0.12	2	0	2
*rpo*C1	2070	99.99	99.95	0.1	1	0	1
*acc*D	1503	99.99	99.93	0.1	1	1	0
*psa*A	2253	99.99	99.96	0.09	1	0	1
*ycf*1	5031	99.99	99.94	0.07	3	1	2
*rpo*C2	4164	99.99	99.92	0.07	3	2	1
*mat*K	1518	99.99	99.93	0.03	1	1	0
*ndh*F	2226	99.99	99.96	0.02	1	1	0
