# Supplementary material for: A comparative analysis of chloroplast genomes revealed the chloroplast heteroplasmy of Artemisia annua
Source: Front Pharmacol. 2024 Aug 14;15:1466578. doi: 10.3389/fphar.2024.1466578 (PMC11349571; doi:10.3389/fphar.2024.1466578)
Supplement: Supplementary file 2 [file DataSheet1.pdf]

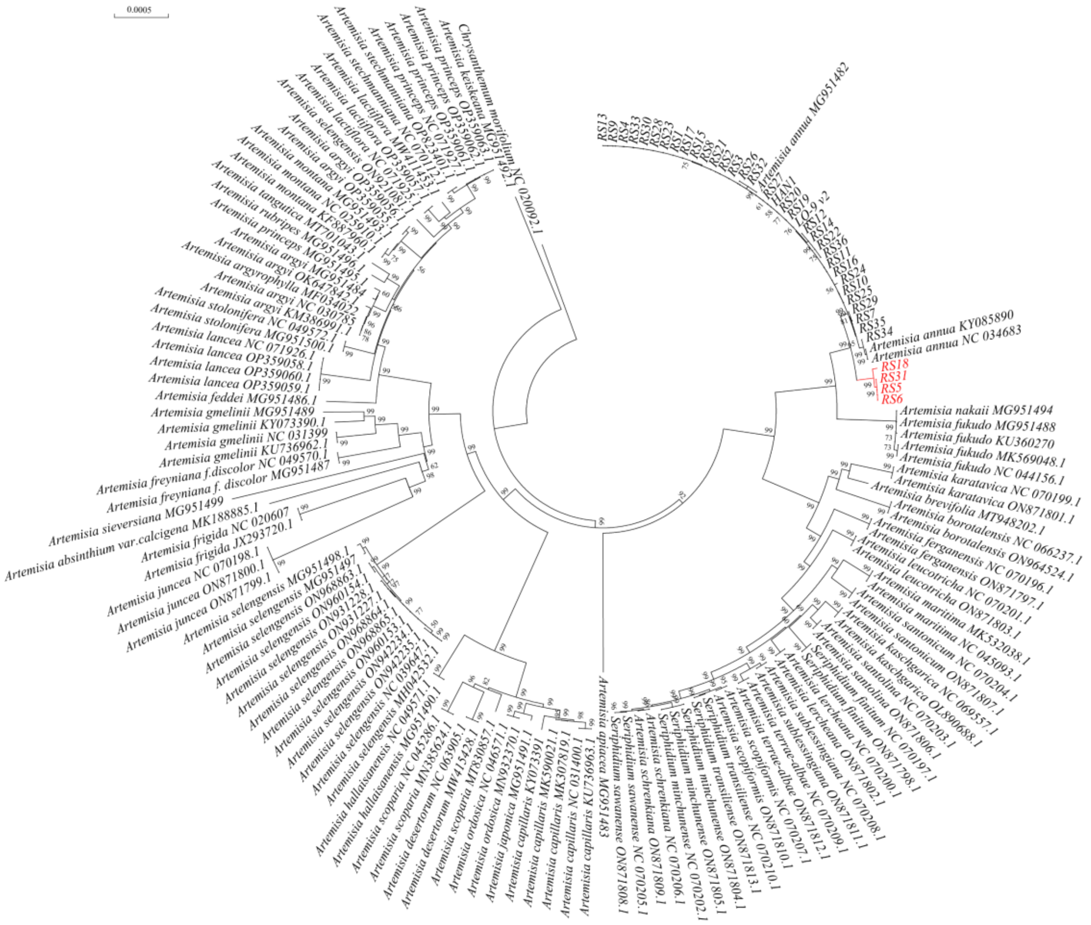

Figure S1 Phylogenetic tree obtained using NJ method for *Artemisia* species based on 156 complete chloroplast genomes. Numbers above the branches indicate the NJ bootstrap values.

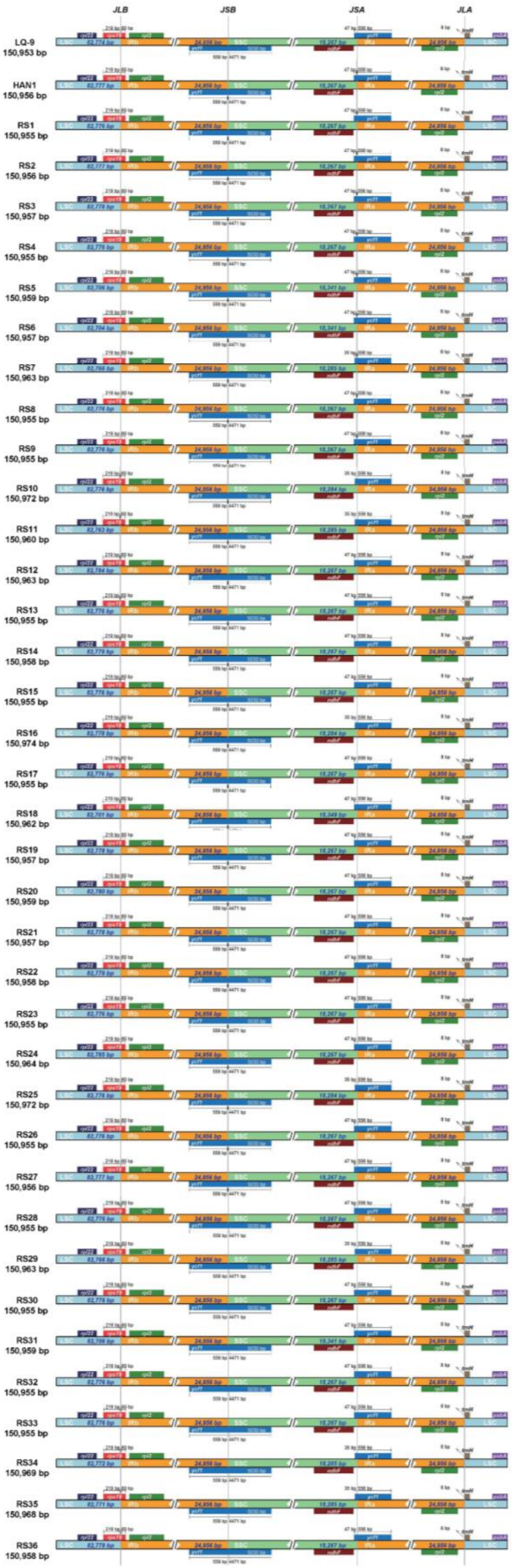

Figure S2 Comparison of the boundaries of LSC, SSC, and IR regions among 38 *A. annua* cp genomes.

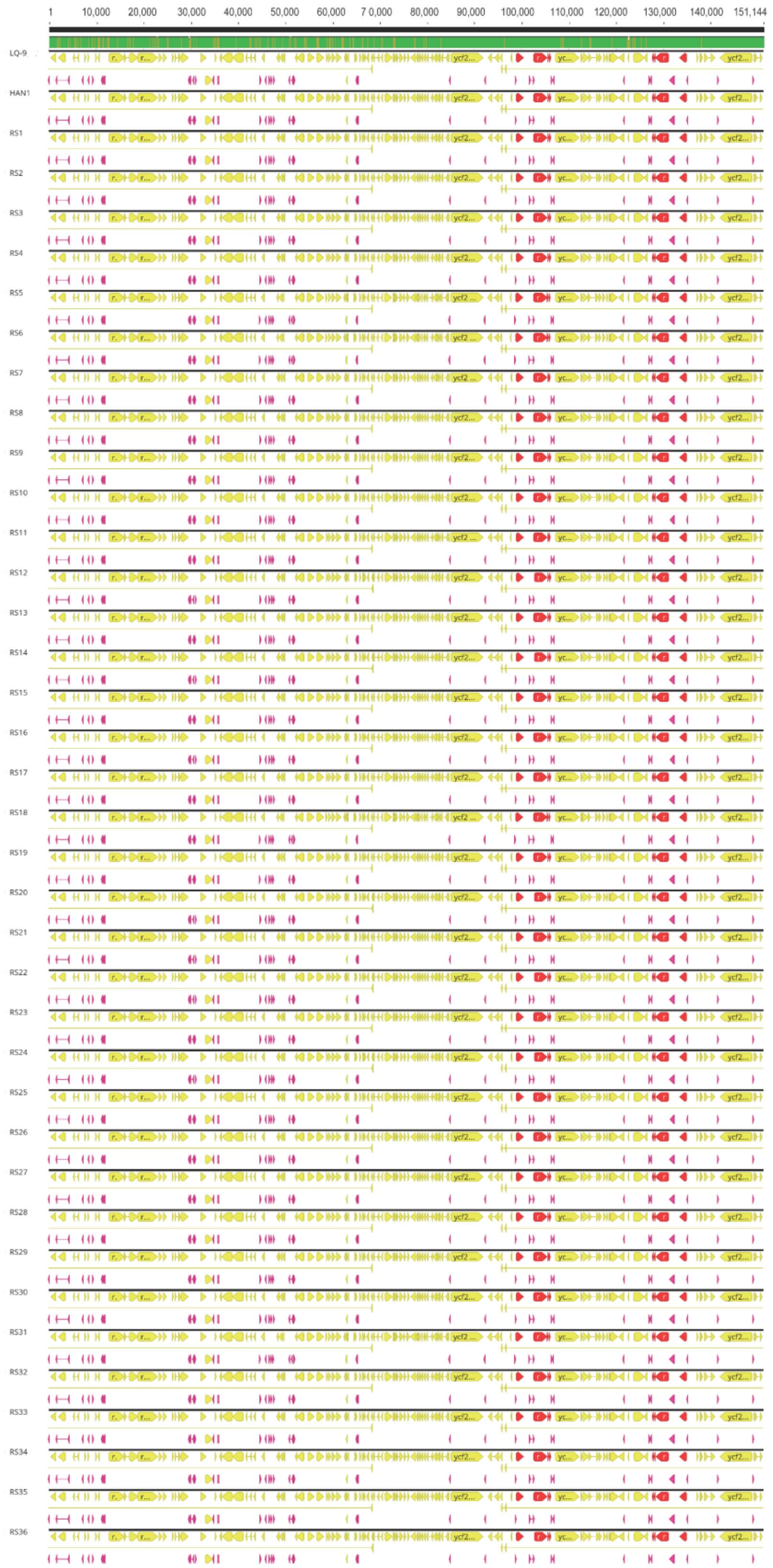

Figure S3 Comparison of genome structure of 38 *A. annua* individuals using Geneious Prime. Annotations of CDSs, tRNA genes, and rRNA genes are shown in yellow, in red, and with purple-red arrowheads, respectively.

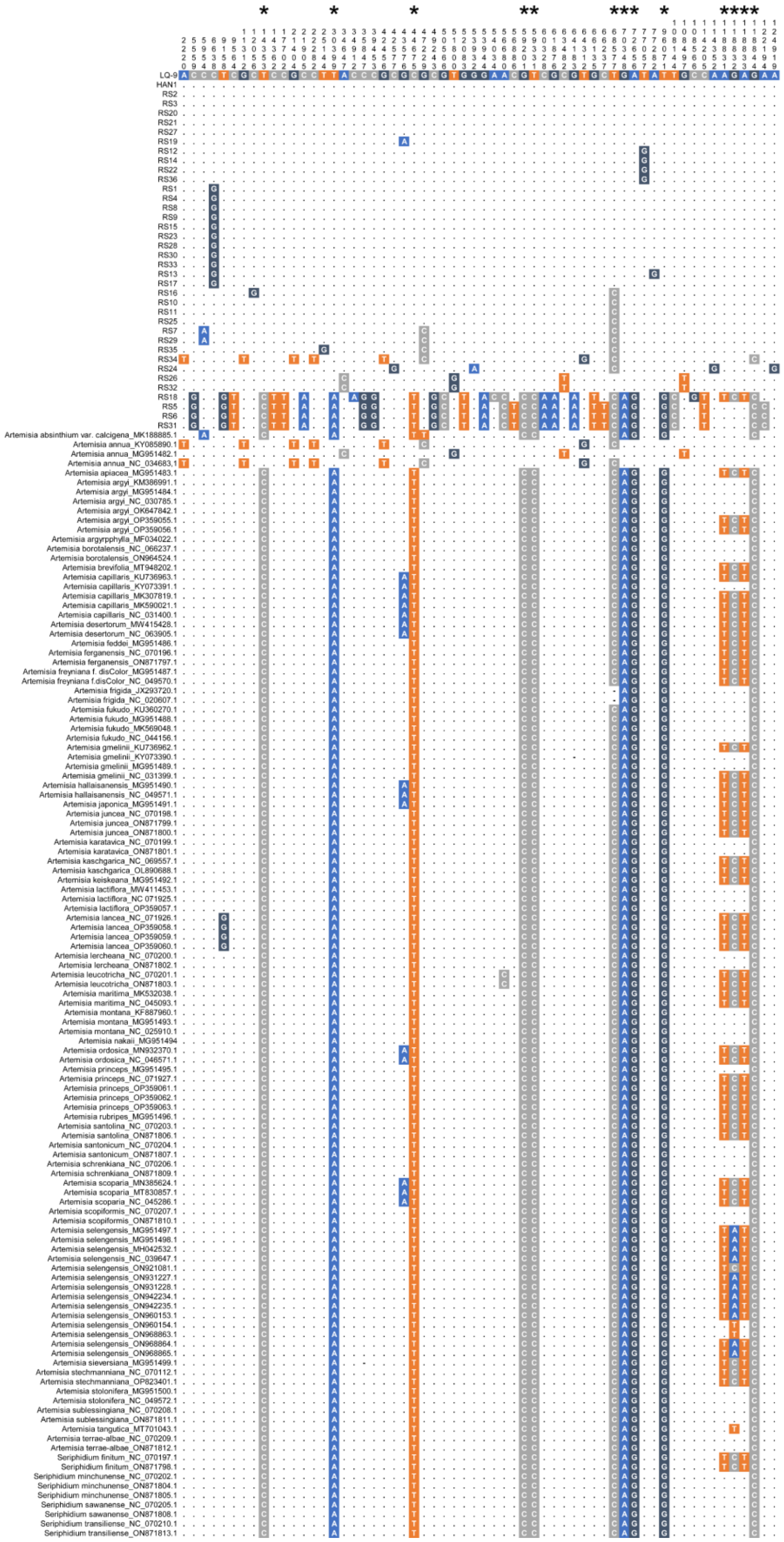

Figure S4 Polymorphism analysis of 38 *Artemisia* species based on *A. annua* 60 polymorphic sites. Polymorphic sites common to most *Artemisia* species are marked in asterisk.
